# Acute Caffeine Mouth Rinse Does Not Change the Hydration Status following a 10 km Run in Recreationally Trained Runners

**DOI:** 10.1155/2020/6598753

**Published:** 2020-06-06

**Authors:** Adam M. Gonzalez, Victoria Guimarães, Nayra Figueiredo, Marcela Queiroz, Paulo Gentil, João F. Mota, Gustavo D. Pimentel

**Affiliations:** ^1^Department of Health Professions, Hofstra University, Hempstead, NY, USA; ^2^Clinical and Sports Nutrition Research Laboratory (Labince), Faculty of Nutrition, Federal University of Goias, Goiânia, GO, Brazil; ^3^College of Physical Education and Dance, Federal University of Goias, Goiania, GO, Brazil

## Abstract

**Background and Aims:**

Caffeine mouth rinsing has emerged as an alternative to oral caffeine consumption for improving performance without provoking lower gastrointestinal distress. However, it remains unclear if hydration status and sweat rate are negatively affected by caffeine mouth rinsing. This study is aimed at evaluating the effects of 10 seconds of caffeine mouth rinsing (1.2% anhydrous caffeine solution) on hydration status and sweat rate following a 10 km run trial.

**Methods:**

Ten recreationally trained runners (30.1 ± 6.4 y) volunteered to participate in this double-blind, placebo-controlled, and crossover research study. Participants completed two 10 km run trials separated by approximately one week. Immediately prior to running, participants completed a 10-second mouth rinse protocol with either 300 mg of caffeine or microcrystalline cellulose (placebo) diluted in 25 mL of water. The effects of caffeine mouth rinsing on hydration status and sweat rate were assessed following a 10 km run trial.

**Results:**

Sweat rate (placebo: 15.34 ± 9.71 vs. caffeine: 11.91 ± 6.98 mL · min^−1^; *p* = 0.39), dehydration (placebo: 1.20 ± 0.57 vs. caffeine: 1.49 ± 0.29%; *p* = 0.15), and hydration (placebo: 15.32 ± 9.71 vs. caffeine: 11.89 ± 6.99 mL · min^−1^; *p* = 0.37) measures were not significantly different between trials.

**Conclusion:**

Caffeine mouth rinse does not appear to alter the hydration status or sweat rate following a 10 km run.

## 1. Introduction

Caffeine remains among the most widely used supplements to improve performance among athletic populations [1]. Serving as a central nervous system stimulant, caffeine acts primarily through the blockade of central and peripheral adenosine receptors [2]. Caffeine-containing supplements are well-known to exert both favorable physical and mental outcomes including increasing exercise performance, as well as enhancing alertness and mental focus [3, 4]. Specifically, caffeine ingestion has shown to play ergogenic effects on endurance capacity and time trial performance in well-trained and recreational subjects [5–7]. However, some negative side effects of caffeine have been reported, especially at high doses [2, 8]. Prior to exercise, caffeine may lead to gastrointestinal distress [9], symptoms of diarrhea [10], and/or diuresis [11]. This process appears to occur via an increase in distal colon motility and gut fluid secretion, along with the inhibition of sodium reabsorption at the level of renal proximal tubules [11, 12]. However, a recent review has concluded that caffeine only exerts a minor diuretic effect which appears to be negated by exercise [13].

Recently, caffeine mouth rinsing has emerged as an alternative to oral caffeine consumption for improving performance without provoking lower gastrointestinal distress [10]. Caffeine mouth rinsing for 5-20 s may elicit ergogenic effects by competitively inhibiting adenosine via binding to adenosine receptors located in the mouth [14]. Additionally, caffeine mouth rinsing may allow for more rapid absorption through the buccal mucosa compared to capsule ingestion [15]. The evidence surrounding the ergogenic effects of caffeine mouth rinsing and aerobic exercise performance is currently equivocal [14]. Sinclair and Bottoms (2014) demonstrated that caffeine mouth rinsing (0.032% caffeine solution) for 5 s immediately before and every 6 min throughout a 30 min arm crank time-trial significantly improved distance covered, power output, and revolutions per min. Similarly, Bottoms et al. (2014) found that caffeine mouth rinsing for 5 seconds with a solution containing 32 mg of caffeine improved 30 min cycling performance via improvements in cadence, power, and velocity. However, a majority of the literature has failed to indicate an ergogenic effect of caffeine mouth rinsing for 5-20 s on aerobic performance [16–19].

Caffeine has been postulated to adversely impact hydration status during exercise performance due to its mild diuretic properties at rest [11]. While the underlying mechanisms have not yet been clearly defined, it has been purported that caffeine can inhibit phosphodiesterases in the proximal tubule of the kidneys [20], and the antagonism of adenosine receptors may also induce mild diuretic properties [21]. Currently, the effect of caffeine mouth rinsing on markers of hydration status following exercise remains unclear. Therefore, the purpose of this study was to investigate the effects of caffeine mouth rinsing on hydration status and sweat rate following a 10 km run in recreationally trained runners. We hypothesized that caffeine mouth rinse would not adversely affect hydration status or sweat rate following a 10 km run.

## 2. Materials and Methods

**Figure 1:**
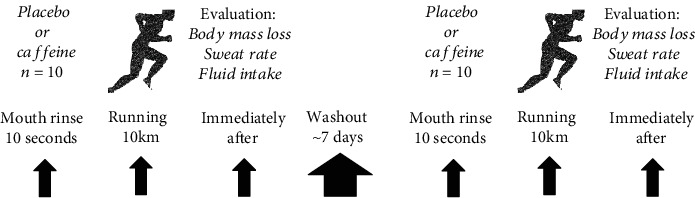
Schematic diagram of the study protocol.

This study was designed as a double-blind, placebo-controlled, and crossover research study. Participants completed two 10 km run trials separated by approximately one week of washout. Immediately prior to running, participants completed a 10-second mouth rinse protocol with either 300 mg of caffeine or microcrystalline cellulose (placebo) diluted in 25 mL of water. The effects of caffeine mouth rinsing on hydration status and sweat rate were assessed following a 10 km run trial. The study protocol is depicted in Figure 1.

Ten recreationally trained male (*n* = 8, 30.0 ± 7.1 y, 1.73 ± 8.0 m, 72.9 ± 9.5 kg, 19.2 ± 8.6% body fat) and female (*n* = 2, 30.5 ± 3.5 y, 1.63 ± 1.0 m, 56.9 ± 1.3 kg, 19.9 ± 5.8% body fat) runners volunteered to participate in this research study. Participants engaged in running exercise between 4 and 7 days per week, completing between 5 and 40 km per week (median: 10.00 km per week). All participants were recreational runners and were not competitive athletes. Inclusion criteria included being a recreational runner and a regular caffeine user; being free of any physical limitations or chronic illness that may affect performance; and not currently using any other medication or performance-enhancing substances. Participants were excluded if they did not meet all of these criteria as determined by a health and activity questionnaire.

Following an explanation of all procedures, risks, and benefits, each participant provided their written informed consent prior to participation in this study. The research was conducted according to the Declaration of Helsinki, and the research protocol was approved by the Federal University of Goias prior to participation enrollment.

The mouth rinse protocol was performed with 300 mg of caffeine (1.2% caffeine solution) or microcrystalline cellulose (placebo) diluted in 25 mL of water. The placebo was indistinguishable in appearance. The caffeine and placebo mouth rinses were separated and labeled by an outside researcher to allow the double-blind design study. Caffeine dosage is consistent with previous studies, which have used 1.2% caffeine solution [18, 22, 23]. All participants were instructed to brush their teeth thirty minutes prior to the mouth rinse protocol. A 10-second mouth rinse protocol was performed immediately prior to initiating the 10 km run. A single 10-second mouth rinse protocol has previously been implemented prior to exercise [22]. Recommendations for the mouth rinse intervention were provided to the participant using previously described methods [24, 25]. Briefly, participants were asked to mouth rinse with either the caffeine or placebo solution for 10 seconds and then expectorate the solution back into a plastic cup.

Participants performed the 10 km run on an outdoor 400 m Olympic race track at the same time of day during the afternoon hours to control for circadian rhythm effects [26]. Participants were instructed to maintain regular water and food consumption within the hours preceding the test, and water was provided ad libitum and recorded during the run. Participants were instructed to complete the race “as quickly as possible.” No feedback was provided to the participant regarding times or heart rates during the run.

Weather conditions, including temperature and humidity, were assessed using a thermometer to identify the possible impact on markers of hydration status. The mean temperature and relative humidity of the air were taken 30 minutes prior to each trial (Day 1: 28.7°C, 59.5%; Day 2: 28.5°C, 56.5%), with no statistical differences (*p* > 0.05) between trials.

Baseline body composition and height were evaluated via dual-energy X-ray absorptiometry (GE Healthcare®, Australia, New Zealand) and a stadiometer, respectively, according to manufacturers' guidelines. Body weight was assessed before the mouth rinse protocol and immediately following the 10 km run using an electronic scale to the nearest ±50 g (Filizola®, São Paulo, SP, Brazil). All runners received a bottle with a measuring scale to quantify total water intake during the run. Hydration status and sweat rate were calculated using the following three equations proposed by Perrella et al. (2005) [27]:

1. *Sweating rate equation* (mL · min^−1^): [(baseline body weight in kg − final body weight in kg) + (water consumed in mL)]/(time to complete the run in minutes)
2. *Dehydration equation* (%): [(baseline body weight in kg − final body weight in kg)∗100]/(baseline body weight in kg)
3. *Hydration equation* (mL · min^−1^): (water consumed in mL)/(time to complete the run in minutes)

Prior to experimental trials, participants completed a 24 h dietary recall to establish habitual food intake. Additionally, habitual frequency of caffeine consumption was obtained using a caffeine-adapted questionnaire [28]. Participants were instructed to maintain their habitual food intake and hydration strategies leading up to the experimental trials. In addition, participants were asked to abstain from caffeine and to avoid physical exercise (other than normal daily activities) for two days prior to each experimental trial.

Initially, a priori analysis of sample size was determined based on data from a previous study [23] and revealed that a minimum of 8 participants were necessary for this study (G-Power software, version 3.1.2, Germany). All statistical analyses were performed by MedCalc® software. Prior to statistical procedures, all data was assessed for normal distribution, homogeneity of variance, and sphericity. Separate paired-samples *t*-tests were also used to evaluate the effect of the mouth rinse protocol on sweat rate, dehydration, and hydration. All between-trials analyses were further assessed using Cohen's *d*. Significance was defined as *p* ≤ 0.05, and all data are reported as mean ± standard deviation.

## 3. Results

Participants' habitual food intake was 2033.8 ± 723.7 calories, consisting of 44.4 ± 8.7% carbohydrate, 27.1 ± 7.7% fat, and 28.0 ± 5.7% protein. Based on the caffeine-adapted questionnaire, participants consumed 24.9 ± 17.2 servings of caffeine per week.

**Figure 2:**
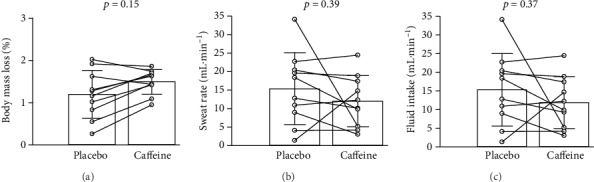
Dehydration, sweating rate, and hydration following the 10 km run.

Dehydration, sweating rate, and hydration are depicted in Figure 2. Dehydration (placebo: 1.20 ± 0.57 vs. caffeine: 1.49 ± 0.29%; *p* = 0.15, *d* = 0.64), sweat rate (placebo: 15.34 ± 9.71*vs.* caffeine: 11.91 ± 6.98 mL · min^−1^; *p* = 0.39, *d* = 0.40), and hydration (placebo: 15.32 ± 9.71*vs.* caffeine: 11.89 ± 6.99 mL · min^−1^; *p* = 0.37, *d* = 0.40) were not significantly different between trials. In addition, no significant differences were noted for total water ingested during experimental trials (placebo: 720.0 ± 437.2 mL*vs.* caffeine: 555.0 ± 326.1 mL, *p* = 0.37).

## 4. Discussion

The objective of this study was to evaluate the effects of a 10-second caffeine mouth rinse protocol on hydration status and sweat rate following a 10 km run trial. To the authors' knowledge, this is the first study to investigate the effects of caffeine mouth rinsing on markers of hydration. The main finding of the present study was that sweat rate and hydration status were not altered by the use of a caffeine mouth rinse protocol compared to a placebo mouth rinse. Our findings are in agreement with a recent meta-analysis showing that caffeine-mediated diuresis does not represent a concern during physical activity [13].

Caffeine is generally recognized as having a mild diuretic effect, and concerns about fluid deficits associated with caffeine ingestion are highly relevant to recreational and competitive athletes [13]. However, several studies have shown that oral administration of caffeine (3-9 mg·kg^−1^) has no effect on core temperature response during physical activity [2]. Additionally, oral administration of caffeine does not influence sweat rate or fluid-electrolyte balance during exercise in the heat [29–32]. Silva et al. (2013) [33] also demonstrated that caffeine ingestion (5 mg · kg^−1^) does not modify extracellular and intracellular fluid distribution, regardless of body composition, physical activity, or daily water ingestion. Currently, there is little evidence to suggest that caffeine is harmful during exercise, even in hot environments [2]. The results of our study are in agreement, indicating that caffeine mouth rinsing also does not negatively impact measures of hydration status. Therefore, the use of caffeine-containing products, including caffeine mouth rinsing protocols, does not need to be avoided prior to exercise due to fear of dehydration.

While our study shows no detrimental effects on hydration, the utility of caffeine mouth rinsing for improving aerobic exercise remains questionable. It has been hypothesized that delivering caffeine in the form of mouth rinse or a chewing gum may speed the rate of caffeine delivery to the blood by absorption through the buccal mucosa [15]. Kamimori et al. (2002) demonstrated faster times to reach maximal caffeine concentrations in caffeine gum trials compared to capsule trials; however, the concentration-time curves did not differ between trials. Nevertheless, Doering et al. (2014) reported no elevation in plasma caffeine during a cycling time trial following 10 seconds of caffeine mouth rinsing. As an alternative mechanism of action, it has also been speculated that caffeine mouth rinsing may competitively inhibit adenosine through direct binding to adenosine receptors located in the mouth and/or through activation of bitter taste receptors located in the oropharyngeal epithelia which have been shown to stimulate regions of the brain associated with information processing and reward [14]. Nevertheless, the ergogenic effects of caffeine mouth rinsing on cognitive performance, high-intensity repeated cycle sprinting, and aerobic exercise are mixed [14]. While some studies have shown improved endurance performance and power output following caffeine mouth rinse protocols [34, 35], the majority of the literature indicates no ergogenic effect of caffeine mouth rinsing on aerobic exercise performance [16–19]. Therefore, more research is needed to fully elucidate the mechanisms by which caffeine mouth rinsing may potentially improve cognition and exercise performance.

The present study is inherently limited by the small sample size and recreational training status of the participants. Additionally, all participants were habitual caffeine users who reported frequent ingestion of caffeine-containing products. Although it is unlikely a major contributing factor, habitual ingestion of caffeine may prevent diuresis, particularly when caffeine is consumed in high doses [36]. Additionally, the bitter taste of caffeine compared to microcrystalline cellulose could have affected the participants' blind effect of study. Although we are unable to measure the VO2max, these participants are recreational runners. Lastly, we did not show the ergogenic effect of caffeine mouth rinsing, since the mean times of the 10 km run were not recorded.

Our findings suggest that caffeine mouth rinsing does not exaggerate fluid loss as indicated by sweat rate and hydration status following a 10 km run trial. Caffeine mouth rinsing appears to be a safe ergogenic aid that can be used by athletes without concerns for any negative impact on fluid balance. Future research should continue to examine the effects of caffeine mouth rinsing on exercise performance. Additionally, further investigation is warranted on different caffeine mouth rinse durations and the effect on plasma caffeine pharmacokinetics and absorption through the buccal mucosa.

## Data Availability

The data used to support the findings of this study are available from the corresponding author upon request.
